# Key issues for establishing a robotics laboratory in the pharmaceutical industry

**DOI:** 10.1155/S1463924694000106

**Published:** 1994

**Authors:** Steve Conder

**Affiliations:** Bristol-Myers Squibb Company PO Box 191 New Brunswick New Jersey 08903-0191 USA

## Abstract

The Analytical Research and Development Department of Bristol-Myers Squibb has a laboratory dedicated to robotic analysis of solid dose forms. It consists of eight individuals responsible for nine robotic systems. The laboratory is dedicated to the support of Phase III stability studies that require dissolution, potency, content. uniformity and Karl Fischer moisture assays. The group performs about 15000 assays a year for approximately six long-term stability programs. The key issues for success were personnel selection, methods development (methods transfer), routine assay support, documentation, validation, training and support services. This paper discusses the establishment of the laboratory and the future issues important to continued success.


Journal of Automatic Chemistry, Vol. 16, No. 4 (July-August 1994), pp. 117-119

### Key issues for establishing a robotics laboratory in the pharmaceutical industry

**Steve Conder**
*Bristol-Myers Squibb Company, PO Box 191, New Brunswick, New Jersey 08903-0191, USA*

*The Analytical Research and Development Department of Bristol-Myers Squibb has a laboratory dedicated to robotic analysis of solid dose forms. It consists of eight individuals responsible for nine robotic systems. The laboratory is dedicated to the support of Phase III stability studies that require dissolution, potency, content uniformity and Karl Fischer moisture assays. The group performs about 15 000 assays a year for approximately six long-term stability programs. The key issues for success were personnel selection, methods development (methods transfer), routine assay support, documentation, validation, training and support services. This paper discusses the establishment of the laboratory and the future issues important to continued success.*

### Introduction

The Analytical Research & Development Department of the Bristol-Myers Squibb (BMS) Pharmaceutical Research Institute has developed a robotics laboratory to provide automated assay support of its Phase III clinical and stability programes within pharmaceutical development.

The typical assays within pharmaceutical analysis that constitute the majority of the work in a Phase III stability programme are:

(1) Potency/degradants.

(2) Dissolution.

(3) Karl Fischer moisture.

The routine Phase III sample load can often exceed the resources available within the non-automated laboratory and the robotics laboratory at BMS has been designed to provide automated support for each of these assays.

The robotics laboratory houses nine robots, two workstations and has a staff of eight (including the laboratory manager). The majority of the robots are dissolution due to the labour intensive nature of this assay, while the remainder are predominantly tablet assay robots or workstations. One robot is used for all Karl Fischer moisture determinations. The laboratory includes:

(1) Nine Robots (Zymark Corporation): five dissolution; three tablet assay; and one Karl Fischer Titration.

This paper was presented at the 1993 International Symposium on Laboratory Automation and Robotics (ISLAR) organized by the Zymark Corporation.

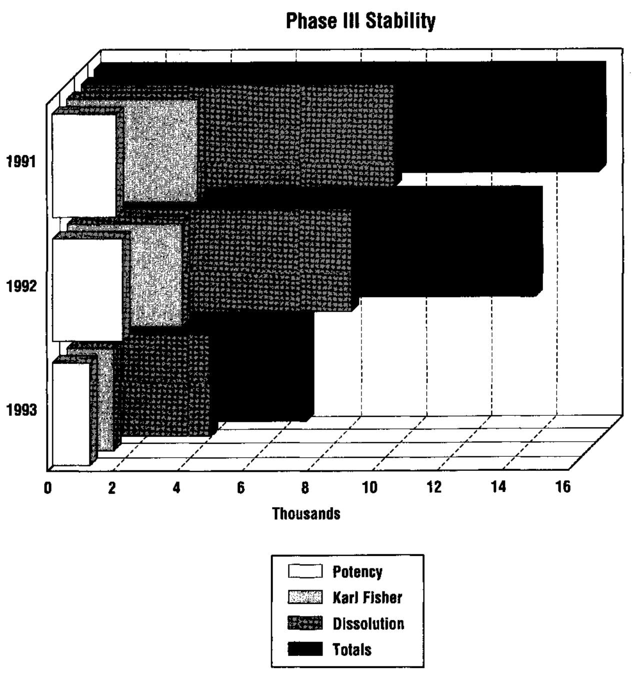

*Figure 1. Sample handling steps.*

(2) Two workstations: BenchMate (Zymark Corporation); and SmartPREP (Source for Automation).

(3) Personnel: four system managers; and three chemical technicians.

A history of the productivity of the laboratory over the past three years is provided in figure 1. The laboratory assayed over 15 000 samples in 1991 of which the majority were dissolution assays. The totals have decreased steadily over the past two years due to the reduced sample loads for older Phase III programmes and the lack of new Phase III programmes that were started during this time. The 15 000 samples assayed in 1991 represent less than 50% of the laboratory's capacity, and there is significant room for expansion.

### Key issues for success

Before discussing the key issues necessary for success in developing a robotics laboratory, it is instructive to review some of the inherent obstacles to success. Several obstacles are presented in table 1 that are worth mentioning here. The first two acknowledge the high cost and demand on resources that an automated laboratory requires, especially during the initial phase. This inevitably leads to special needs for education of the users or system managers for each robotic system. Furthermore, the complexity of robotic systems for total automation of the

© Zymark Corporation 1994

*Table 1. Inherent Obstacles to success.*

| |
|---|
| High cost |
| High demand for resources |
| User education |
| Complexity |
| Long automation design cycle |
| GLP/GMP requirements |

*Table 2. Key issues for success.*

| |
|---|
| Personnel selection |
| Methods development |
| Routine assay support |
| Training |
| Documentation/validation |
| Support services |

typical assays can lead to extended design cycles. Rarely is success achieved immediately upon installation. Finally, a special requirement particular to the Pharmaceutical Industry is the need for adherence to current Good Laboratory Practices (GLP) and Good Manufacturing Practices (GMP) regulations within the operating laboratories.

The key issues for success are shown in table 2. The assumption here is that management support and adequate funding and facilities are available for the laboratory.

The most important factor is personnel selection. Many obstacles or inadequate support in the other areas listed in table 1 can be overcome by a creative and highly motivated staff. The ideal candidate has strong analytical skills, is computer literate and is detail oriented. Understanding the key steps in an analytical method is essential to its successful automation. Successful automation chemists are also part engineer (both mechanical and software) since they perform the translation of the manual procedure into an automated one to be performed by a blind, deaf and dumb robot. This can be a frustrating experience that requires perserverance, creativity and a willingness to experiment and question assumptions. However, perfectionist tendencies can be fatal because at some point development steps and routine samples need to be run.

A key area for success in assay support is methods development. Experience has taught BMS to follow an experimental plan that has developed over time for each assay type that is automated. Initially, this did not exist as experience was gained about the importance of certain assay requirements. For automation of manual dissolution assays, the appropriateness of the method for use on the robot is assessed and, if suitable, the validation plan is developed to transfer the assay. This allows templates to be built which are followed during method development and speeds the whole process to completion. This is particularly important for stability assays where it is necessary to make the transition from development to heavy support for routine assays very quickly and smoothly. It is critical to develop efficient procedures for handling sample and data management very early in the development cycle. This is necessary to avoid the classic automation bottleneck that occurs when robots stand idle while results are calculated and notebook entries are completed.

A tedious and time-consuming task that can be viewed as unproductive is the GLP/GMP validation and documentation of a system. The documentation is comprised of a considerable volume of paper on the hardware and software that constitutes a robotic system. This is not provided with most installations and must be prepared by the user. Validation requires an understanding of the key procedures or processes under the control of the robot that must be tested to ensure accuracy, suitability and adherence to any GMP requirements. This may be as simple as validating the determination of the vessel temperature in a dissolution test that must meet USP criteria. Since it is only recently that this information can be provided by the manufacturer, the users generally decided what documentation was necessary. Furtheremore, provisions for system security and software change control need to be addressed as a part of computer system validation.

Technical support services are needed both from the vendor and in-house to adequately service, maintain and integrate the robotic system within the data management systems of the development. Robots that perform LC injections can create a heavy workload for a chromatographic data processing system; it is a key requirement to have sufficient capacity for each robot. Furthermore, development of custom assay methodologies may require engineering services, such as mechnical or electrical to develop custom hardware for each application. Once again, this is a labour intensive and iterative process.

### Future plans

BMS has shifted from late stage development projects with heavy Phase III support requirements to early stage projects that have vastly different dynamics than Phase III programmes. Experiences with Phase III programs has provided a substantial amount of information about methods development strategies and sample/data management for high-load projects. However, to participate and contribute to early stage development projects, it is important to be flexible and expand the laboratory's capabilities. For example, early stage development dose forms are typically dry-filled capsules that pose different challenges for dissolution and potency assays than tablets. It is necessary to rely on our methods development strategies to respond quickly and continue to expand the laboratory's technological capability to assay dry-filled capsules. Furthermore, it demands more of the laboratory's analytical capabilities, since less is known about the compound and dose form behavior. The ability to automate at an early stage will be a bonus if the project moves to the latter stages of development, since a robot-friendly analytical method is available.

**Acknowledgements**

The author would like to thank the following for their valuable contributions to the success of this project. Current and past management of ARD including: Drs Berry J. Kline, Glenn A. Brewer and Jerry R. Allison. The current members of the robotics laboratory team: Dan Barrow, Scott Jennings, Rich Vol Culin, John Rumney, Jim Wysocki, Alma Johnson and Khanh Ha.

